# Graduate Study on the Continent—Germany

**Published:** 1908-09-19

**Authors:** 


### HOSPITAL ADMINISTRATION.

### CONSTRUCTION AND ECONOMICS.

### GRADUATE STUDY ON THE CONTINENT—GERMANY.

X.—STRASBURG AND FREIBURG.

It will have been noticed in the preceding articles that we have not by any means kept ourselves to a strictly geographical classification of German centres for post-graduate study. We have jumped, so to speak, from the north Prussian centres to the heart of Bavaria, from the extreme east, Breslau, to the extreme west, Cologne. Nor has this been done without an object, namely, to give the student a broad outline of the smaller clinical centres, irrespective of their geographical position, leaving the larger and more important towns to be dealt with later on. Thus we have thought it advisable to deal specially with Munich and Leipsig, both centres which enjoy a deserved and well-recognised reputation, and it remains now to deal with two less well known (to English graduates at least), but in our opinion no less interesting and important centres, Strasburg and Freiburg.

#### Strasburg.

The "Kaiser Wilhelm" University of Strasburg was founded in the year 1567, and reorganised in 1872, after the war with France. The matriculation fee is 20 marks, and all applications must be handed in during the first four weeks of any academical term. Guest tickets are issued on peculiar conditions, differing somewhat from those that obtain in other University towns. Women students, for instance, are not admitted to "asterisked lectures," that is to say to those marked in the calendar with an asterisk, but each professor or privat docent has the right to admit or refuse admission to any lady student to his lectures or demonstrations. Guests must be, for the time being at least, domiciled in Strasburg. (This regulation is by no means exacted, and for all practical purposes a day's stay at a hotel will count as "domicile.") The usual students' fees are required, and there are various prizes and scholarships, open only to active students who have attended lectures for several semesters and are German subjects. Living is on the whole cheap, but the lodgings cannot be compared with those obtainable in Berlin or Tubingen at the same price. They average 25 marks per month, heating and attendance being separately charged for. Good midday meals may be obtained at any of the restaurants frequented by students, at prices ranging from 15-40 marks monthly. The pensions are good and cheap. Hotels, on the other hand, are expensive and not to be recommended.

The medical faculty at Strasburg is a strong, though not a large, one. It includes such teachers as Professors von Recklinghausen (pathological anatomy), Schmiedeberg (toxicology), Madelung (surgery), Ewald (physiology), Fehling (gynæcology), Wollenberg (psychiatry), Naunyn (medicine), Stilling (ophthalmology), Wolff (dermatology), Bayer (midwifery), Manasse (otology), and Levy (bacteriology), while among the privat docenten one finds such well-known names as those of Klein (gynæcology), Bethe (psycho-physiology), Landolt (ophthalmology), Rosenfeld (forensic medicine), Schlesinger (pediatrics), and Fuchs (embryology). The various clinical institutions are all worth a visit, though many of them are not of the newest. Specially to be mentioned are the fine medical clinic under charge of Professor Moritz, the Institut für Hygiene und Bakteriologie, under Professors Forster and Levy; the Frauen Klinik, under Professor Fehling, and the fine surgical clinic where Professor Madelung operates daily. The professors give regular courses for graduate students (usually limited to private practitioners residing in Elsace Lorraine, application for admission to be made directly to the demonstrators), while private courses can be obtained as well. At the out-patients' French as well as German is spoken, and the student who wishes to remain for any length of time at Strasburg will find it advisable to brush up his knowledge of French. German is, of course, the official language, and most of the lectures and demonstrations are given in German. There is a small but fairly well stocked library for medical students, while the usual institutes for the preliminary and ancillary subjects are up to date and good.

#### Freiburg.

It would be difficult to find a German University that can compete with this little town in Baden as far as the reputation of its medical faculty and its value as a medical school is concerned. Among the professors one finds Professors Manz (ophthalmology), Hegar (gynæcology), Hildebrand (botany), Bäumber (medicine), Kraske (surgery), Schottelius (hygiene), Killiani (chemical physiology), Axenfeld (ophthalmology), Hoche (psychiatry), Aschoff (pathology), Killian (laryngology), Jacobi (dermatology), Bloch (otology), Goldmann (surgery), Fischer (anthropology), and Roos (balneotherapy). The clinics, though comparatively small, are very interesting, and the material is ample when one considers that Freiburg is not overcrowded with students. It is in some respects an ideal "small centre." Lying far away from the distractions of the large cities, it is yet conveniently close to these to make a sojourn no exile for the graduate who feels himself too much penned up in a small University town. The surroundings are fine, and the climate is throughout preferable to that of a more northern centre. Nor are the social attractions of Freiburg few or far between. It has its theatre and its concert hall, and although it cannot boast to possess an English colony in the same sense as Heidelburg or Munich possesses one, there are yet a large number of foreigners resident in the town. To English graduates Freiburg is far less well known than it deserves to be, and it is a centre that we can thoroughly recommend. The Albert-Ludwig University is one of the oldest in southern Germany, dating back to the year 1457, and opens its gates to women students, as well as to foreign graduates. The matriculation fee is 20 marks, and the conditions for matriculation are similar to those that obtain at other centres. Guest tickets are only exceptionally granted; for a short stay they are by no means necessary, as the graduate is admitted to all lectures and demonstrations on introduction to the professor or docent. There is an excellent University library, and the usual collections of pathological, anatomical, and clinical interest will well repay study.

Living at Freiburg is cheap, comfortable, and good. There are many good pensions and small hotels wherein the visitor will be treated with every courtesy, and in which he will be comfortably housed at prices ranging from 100 to 150 marks monthly. Rooms may be obtained in the neighbourhood of the clinics at a monthly rental of from 20 to 70 marks. Here, as elsewhere, the student is recommended to inquire carefully before taking any room if the monthly charge includes such things as attendance, lighting, heating, and boot-cleaning. Breakfast may be compounded for at a monthly charge of 12 to 15 marks. Other meals are best taken at one of the smaller restaurants, where the food is everywhere good and the prices are moderate. Very fine rooms at a slightly higher rental may be hired in the outskirts of the town, and on the whole these are to be recommended.

Clinically Freiburg is one of the most interesting of the smaller University centres in Germany. No surgical student, for example, should omit to visit Professor Kraske's clinic, or to see Professor Killian's demonstrations with the laryngoscope, the bronchoscope, and the œsophagoscope. Similarly the gynæcological clinics are excellent; while in general surgery Freiburg offers many opportunities for study. The clinics are, as we have already stated, relatively small, but the in- and out-patient material is diversified and rich, and a month's study here will be amply repaid. One sees many interesting cases, and almost daily there is a chance of observing what is relatively a "rare" case, at least in English hospitals. In abdominal surgery, and in surgery of the lungs and thorax especially, Freiburg can easily hold its own with any other European centre. There are practically very few post-graduate students studying here at present, and it is only due to the fact that the place is not so well known as it deserves to be that more do not avail themselves of the exceptional opportunities that are offered.

There is no exaggeration in calling these opportunities "exceptional." When one considers that Freiburg is not a large centre, and that it does not lay claim to being a Mecca, as Vienna, Berlin, and Paris do, one is astonished to find that the graduate student is so well catered for; not only do the teachers, when asked, give private courses, not only are there chances for the foreign graduate (provided he knows German) to work in one of the clinics as assistant or volunteer under men who are recognised experts in their special departments, and not only are there special demonstrations and lectures (such as those in the subject of balneotherapy), but in addition regular vacation courses are given under specially favourable conditions. These courses are open to all practitioners resident in the Grand Duchy of Baden on payment of a fee of 20 marks (£1). This fee admits them to *all* courses, practical and theoretical, and gives them the privilege of attending all clinical demonstrations, lectures, etc., as guests. Foreign graduates and all others not domiciled in Baden must pay this initial fee and in addition a "course fee" of 1s. 6d. (1.50 marks) for each course—really a very small sum when one considers the excellence of the courses. They have the same privileges as the other members. For particulars of the courses graduates should apply to the General Secretary of the Local Committee for Graduate Study in Freiburg, Privat Docent Dr. Link, Freiburg. The President of the Committee is Geheim. Rat. Professor Dr. Bäumler, Universitats Klinik, Freiburg.

Those who have attended the courses speak very highly of them; they are in every way practical, adapted to the needs of the graduate, with limited membership, and solely designed for the benefit of the general practitioner. To those who do not wish to attend courses at a larger centre we can cordially recommend Freiburg as one of the best post-graduate centres to be found in Germany.

### Some Non-University Centres.

So far we have dealt almost solely with centres which are "university centres." There are, however, other towns in Germany which possess excellent clinical material, owing to their fine local hospitals, where the student may find it profitable to remain for a while. It must not be supposed that the university towns alone offer attractions. If we were asked to name the best post-graduate centre in Germany, apart from Berlin, we should have no hesitation whatever in saying Hamburg, and with the excellent courses at Cologne we have already dealt. Want of space forbids us from fully entering into particulars about other non-university centres, but a list of them may not be without value here.

*Altona*: Courses in March and November, chiefly in surgery; Professor König (medicine), Professor Umber (pathology and psychiatry). Local Secretary: Prosektor Dr. Hueter.

*Barmen*: Courses in practically all subjects during the winter from October to March. Local Secretary: Dr. Koll.

*Beuthen*: Similar courses. Local Secretary: Dr. Born.

*Danzig*: Winter and summer courses in all subjects. Local Secretary: Dr. Adolf Wallenberg.

*Düsseldorf*: Excellent summer courses in special subjects. Local Secretary: Professor A. Hoffmann.

*Elberfeld*: Courses in special subjects are held from January to April. Local Secretary: San. Rat. Dr. Kleinschmidt.

*Frankfurt a. M.*: Winter, Christmas, and summer courses. Local Secretary: Dr. Konig.

*Nürnberg*: Very good practical courses are held here during the winter. The clinical material is rich and varied. Local Secretary: Hofrat Dr. Schuh.

*Dresden*: Free courses are held in the winter term. A feature is the course in gynæcology held at the Royal Gynæcological Hospital. Local Secretary: Oberarzt Dr. Kannigiesser.

These courses are all held under the auspices of the Central Committee for Post-Graduate Instruction in Germany (Secretary, Professor R. Kutner, Luizen Platz, Berlin) and are for the most part free. In addition courses are held at a large number of smaller centres. These latter are not, however, to be recommended for the foreign graduate, as, however good they are, the material available is not large and opportunities for individual work are not so frequent as elsewhere.

